# Toward an understanding of real-world mobility in Parkinson’s: insights from enhanced contextualisation using GPS-derived location and data-driven modeling of walking speed

**DOI:** 10.3389/fnagi.2026.1746429

**Published:** 2026-02-20

**Authors:** Cameron Kirk, Rana Zia Ur Rehman, Brook Galna, Saverio Ranciati, Emma Packer, Neil Ireson, Vitaveska Lanfranchi, Claudia Mazzà, Lisa Alcock, Lynn Rochester, Alison J. Yarnall, Silvia Del Din

**Affiliations:** 1 Faculty of Medical Sciences, Translational and Clinical Research Institute, Newcastle University, Newcastle upon Tyne, United Kingdom; 2 Janssen Research and Development, High Wycombe, United Kingdom; 3 School of Allied Health (Exercise Science)/Centre for Healthy Ageing, Health Futures Institute, Murdoch University, Perth, WA, Australia; 4 Department of Statistical Science “Paolo Fortunati”, University of Bologna, Bologna, Italy; 5 Department of Computer Science and INSIGNEO Institute for in silico Medicine, The University of Sheffield, Sheffield, United Kingdom; 6 School of Mechanical, Aerospace and Civil Engineering, The University of Sheffield, Sheffield, United Kingdom; 7 Indivi AG, Basel, Switzerland; 8 National Institute for Health and Care Research (NIHR) Newcastle Biomedical Research Centre (BRC), Newcastle upon Tyne, United Kingdom; 9 Newcastle upon Tyne Hospitals NHS Foundations Trust, Newcastle upon Tyne, United Kingdom

**Keywords:** digital mobility outcomes, Gaussian Mixture Modeling, GPS context, machine learning, Parkinson’s, real-world gait, walking speed, wearable sensors

## Abstract

**Introduction:**

Conventional clinical assessments do not fully capture how Parkinson’s disease (PD) affects mobility in daily life. Integrating digital mobility outcomes (DMOs) from wearable devices with GPS-derived contextual data could provide richer insight into real-world mobility, yet this approach remains largely unexplored. Similarly, data-driven modeling of DMO distributions, such as walking speed, may reveal clinically relevant changes in mobility that are obscured by averaged measures. This study (i) examined how indoor–outdoor context enhances interpretation of real-world mobility, and (ii) applied Gaussian Mixture Modeling (GMM) to characterize data-driven patterns within walking speed distributions in people with PD.

**Methods:**

Fifty-two people with PD (PwP) and 19 older adult controls were recruited from the CiC and Mobilise-D studies. DMOs were estimated from a single wearable device, and indoor-outdoor location was synchronized with GPS data from a smartphone. GMM was applied to estimate the optimal number of walking speed modes. Generalized linear models compared DMOs between indoor and outdoor contexts and between cohorts, adjusting for age and sex.

**Results:**

Thirty-nine PwP and 17 controls had valid contextual data. Both cohorts performed significantly more indoor than outdoor walking bouts, with longer walking durations outdoors. Only controls walked significantly slower and with shorter strides indoors versus outdoors, while both groups showed longer stride duration indoors. Between-cohort differences emerged only outdoors, with PwP exhibiting higher cadence. Most participants across both cohorts displayed three walking speed modes, which were associated with medication dosage and motor severity.

**Discussion:**

This study demonstrates the potential of GPS-derived contextual information to enhance interpretation of real-world mobility outcomes in PD. Walking speed modes show promise for capturing novel clinical insight, though further technical and clinical validation is required to establish their robustness and clinical relevance.

## 1 Introduction

Parkinson’s disease (PD) is a progressive neurological disorder characterized by the cardinal motor symptoms of bradykinesia, rigidity, tremor and postural instability (Jankovic, 2008; Postuma et al., 2015). The Movement Disorder Society-Unified Parkinson’s Disease Rating Scale Part III (MDS-UPDRS III) remains the most widely used method to evaluate motor symptom severity (Goetz et al., 2008). There are several limitations associated with its use; most fundamentally it does not directly assess aspects of health which are of importance to people with Parkinson’s (PwP), such as preservation of their everyday physical mobility (Deane et al., 2014; Delgado-Ortiz et al., 2023; Mammen et al., 2025). Digital mobility outcomes (DMOs), such as walking speed and step count, can be robustly measured in real-world settings with wearables (Kirk et al., 2024b; Micó-Amigo et al., 2023). While wearables have shown promise in complementing existing clinical assessments of PD (Kirk et al., 2025), practical challenges remain for certain devices. For example, some devices consist of multiple sensors, requiring higher sampling frequency, limiting battery life. These may also require re-equipping after accidental removal, and adherence may be more difficult in specific subgroups, such as individuals with cognitive impairment who may forget to consistently wear or charge the device. In addition, a broader challenge across all wearables lies in the contextual interpretation of large, continuously collected datasets

Real-world mobility is assessed across diverse indoor and outdoor environments that place different demands on motor control, such as navigation of tight spaces and obstacles, walking for prolonged periods and negotiating changes in the walking surface from weather (Kim and Brown, 2022; Toda et al., 2020), or terrain (Kowalsky et al., 2021). PwP may choose to walk outdoors primarily during “ON” medication periods (Del Din et al., 2016), while more confined indoor environments may capture freezing of gait (FOG) disturbances (Zhang et al., 2021). Without contextual awareness, it is not clear whether changes in gait parameters are pathological or may instead reflect normal adaptations to environmental conditions (Marcianò et al., 2025). At present the context of real-world DMOs is solely inferred based upon walking bout (WB) thresholds. WBs are defined as walking sequences containing at least two consecutive strides of both feet. The start and end of a WB are determined by a resting period or any other activity (non-walking period) (Kluge et al., 2021). It is speculated that short WBs (<30 s) are more likely to occur indoors and longer WBs (>30 s) over open outdoor spaces. Estimation of context is possible with use of Global Positioning System (GPS) or Global Navigation Satellite System receivers, embedded within specialist devices, smart watches and smart phones, which despite broad applications have yet to be combined with continuous real-world mobility data (Bayat et al., 2020; Breasail et al., 2021; Jankowska et al., 2015). Differentiating between contexts could provide clinicians with richer insight into how mobility limitations manifest in daily life, beyond what can be observed during short, structured in-clinic assessments.

Alongside contextual factors, novel analytical approaches may offer valuable insights into how PwP adapt their walking across real-world environments and medication states (Althoff et al., 2017). DMOs are typically summarized as singular values, which may obscure clinically important patterns within its distribution. Walking speed provides a global measure of mobility, prone to variability from environmental and physiological demands, being influenced by cognitive impairment (Rochester et al., 2014), FOG (Zhang et al., 2021), medication effects (Corrà et al., 2021), and deficits in balance and muscle function (Pääsuke et al., 2004; Pelicioni et al., 2021; Raccagni et al., 2020). This may create a fluctuating pattern in the distribution of walking speed, consisting of multiple modes where slower modes may capture cautious walking or mobility limitations, while faster modes may reflect more optimal performance during “ON” periods or in less challenging environments. A greater number of modes could reflect greater adaptability, or fluctuating pattern influenced by gait-disturbances or medication (Atrsaei et al., 2021; Mancini et al., 2018). Despite its potential, there is currently no standard approach for modeling such patterns, as existing research has focused upon estimating a fixed bimodal distribution of walking speed. Gaussian Mixture Modeling (GMM), an unsupervised machine-learning clustering technique, could objectively estimate the number of walking speed modes within an individual’s distribution potentially providing novel understanding of how PD affects real-world mobility. However, this method has yet to be applied to continuous real-world mobility data, and its clinical utility remains to be established.

The aims of this study were to: (i) investigate the potential of indoor and outdoor contextual location information to enhance understanding of real-world digital mobility outcomes in PwP and control participants and (ii) explore the utility of a novel application of a data-driven machine-learning approach modeling the number of modes within the distribution of walking speed.

## 2 Materials and methods

### 2.1 Participants

PwP were recruited between June 2021 to June 2024 as part of the Medical Research Council (MRC) Confidence in Concept (CiC) funded study “Translating digital healthcare to enhance clinical management: evaluating the effect of medication on mobility in people with Parkinson’s Disease” (Packer et al., 2023). PwP were assessed in Newcastle upon Tyne, United Kingdom. Inclusion criteria were consenting adults aged 18 years or older (no upper limit) diagnosed with PD based on the Movement Disorder criteria (Postuma et al., 2015), H&Y stages I–III (Hoehn and Yahr, 1967). Individuals were also required to be on stable PD medication for 4-weeks before participation. Ethical approval was obtained from the London—Westminster Research Ethics Committee (REC reference: 21/PR/0469) and the study was conducted in accordance with the declaration of Helsinki.

Older Adults control participants (controls) of a similar age were recruited from the Mobilise-D Technical Validation Study (TVS) (Mazzà et al., 2021), which validated a wearable-device based algorithmic pipeline for estimating DMOs across multiple cohorts, assessment conditions and confounding factors. Inclusion criteria comprised age 65 years or older and able to walk independently with or without walking aids. Controls were recruited from four sites based in Israel, Germany and the United Kingdom: Tel Aviv Sourasky Medical Centre, Israel (ethics approval granted by the Helsinki Committee, Tel Aviv Sourasky Medical Centre, Tel Aviv, Israel, 0551-19TLV), Robert Bosch Foundation for Medical Research, Germany (ethics approval granted by the ethical committee of the medical faculty of The University of Tübingen, 647/2019BO2), University of Kiel, Germany (ethics approval granted by the ethical committee of the medical faculty of Kiel University, D438/18) and The Newcastle upon Tyne Hospitals NHS Foundation Trust, UK (ethics approval granted by London—Bloomsbury Research Ethics committee, 19/LO/1507).

### 2.2 Demographic and clinical measures

In addition to demographic characteristics such as age, mass, height etc., data were collected on disease duration and medication use, PwP were assessed with the MDS-UPDRS (Goetz et al., 2008) and Levodopa equivalent daily dosage (LEDD) was calculated (Tomlinson et al., 2010), FOG questionnaire total score (Hulzinga et al., 2020) and the Late-life function disability index (LLFDI) (Haley et al., 2002; Jette et al., 2002).

### 2.3 Real-world gait assessment protocol

#### 2.3.1 Mobility assessment

Each participant was equipped with a wearable device, worn on the fifth lumbar vertebra (L5), and asked to continue their normal routine while being monitored for seven consecutive days. Control participants wore a McRoberts Dynaport MM+ (McRoberts, The Hague, Netherlands) with a Velcro strap, whereas PwP wore an Axivity AX6 device (Axivity Ltd., Newcastle upon Tyne, United Kingdom), attached with a medical-grade hydrogel adhesive patch (Nile, Ängelholm, Sweden) and covered with a Hypafix™ bandage. The two devices were metrologically equivalent (Del Din et al., 2025) and embedded a six Degrees of Freedom (DoF) inertial measurement unit with the following configuration: triaxial accelerometer with a range of ±8 g and a resolution of 1 mg, triaxial gyroscope with a range of ±2,000 deg/s and a resolution of 70 m°/s, sampling frequency 100 Hz. Only participants with > 2 days of data were included in analysis (Buekers et al., 2024).

#### 2.3.2 Contextual factor assessment

The participants were asked to carry a Samsung Galaxy S9, S10, 21 or s23+ (Samsung, Seoul, South Korea) smartphone. GPS information that was needed to estimate indoor and outdoor location was captured by the AEQORA application in the TVS and Mobin application in the CiC study (Ciravegna et al., 2019; Debelle et al., 2023). Indoor and outdoor locations were estimated from stay point information (Ciravegna et al., 2019; Marcianò et al., 2025). Stay point estimation uses the proximate consecutive GPS locations to indicate the participant has stopped at a given location. Stay points were identified as the centroids of spatial and temporal clusters where GPS data are located within a predefined radius of 20 m for over 2 min (or in the absence of data, considering that the app did not report the location within a radius of 10 m from the previous location) (Marcianò et al., 2025). Stay points were then clustered to determine the actual stay points using Hierarchical Density-Based Spatial Clustering (HDBSCAN) algorithm (Ester et al., 1996). Location semantics, such as the nearest building or area of land, were derived from the relevant area of the OpenStreetMap (OSM), over which the GPS coordinates overlap. The data from OSM were used to identify the land use associated with each stay point (parks, forests, urban), and provide a percentage probability that the walking activity is indoors. This probability reflects the strength of available GPS-based evidence rather than a continuous confidence score. A probability value of 0.5 indicates insufficient or absent location evidence, which primarily occurs when the smartphone was inactive and GPS sampling was suspended to minimize battery consumption. In contrast, probabilities deviating from 0.5 indicate sufficient location evidence to infer indoor or outdoor context. Based on this interpretation, probabilities < 0.5 were classified as outdoor walking and probabilities > 0.5 as indoor walking, while values at or near 0.5 reflect uncertainty rather than ambiguous classification. This threshold therefore distinguishes between absence of evidence and evidence-informed location inference, rather than representing a conventional probabilistic decision boundary.

### 2.4 Digital mobility outcome estimation

The sensor data were first standardized according to the protocol outlined in Palmerini et al. (2023). The standardized data is processed by the validated Mobilise-D processing pipeline. The validation of this processing pipeline, based on a minimum 95% CI ICC threshold for performance metrics (i.e., sensitivity, positive predictive value, accuracy etc.) of 0.7 and a relative error of less than 20%, is described in Micó-Amigo et al. (2023) and Kirk et al. (2024b). These algorithms were written in Matlab and Python coding languages and implemented in MATLAB^®^ R2023a (Mathworks, California, United States), alongside Python and Docker Containers (Watson and Hiden, 2022). A complete description of the pipeline is provided in Kirk et al. (2024a), the pipeline consists of blocks with enable identification of gait sequences and of initial contacts and estimation of cadence and stride length. Stride length estimation required participant height, which was included as a parameter within the inverted pendulum model to estimate stride length (Soltani et al., 2021; Zijlstra and Hof, 2003). Walking speed was then estimated from the outputs of stride length and cadence algorithms defined as the ratio of cadence to stride length. Two independent analytical pipelines were applied for the control and PD cohorts due to optimization of gait-sequence detection and cadence algorithms for respective cohorts as reported in Micó-Amigo et al. (2023). Twenty-two DMOs were estimated at a stride and WB level (Kluge et al., 2021) assessing the amount and pattern of walking activity and pace and rhythm of gait.

DMOs were first estimated on a stride level, conforming to consensus agreed definitions for WBs (Kluge et al., 2021). Accordingly, a WB was defined as a continuous sequence containing at least two consecutive strides of both feet (e.g., R–L–R–L–R–L or L–R–L–R–L–R, being R/L the right/left foot making contact with the ground); consecutive WBs were defined if a break greater than 3 s was identified between them; and, for a stride to be included in a given WB it had to have a duration between 0.2 and 3.0 s and a stride length > 0.15 m (Bonci et al., 2020). WBs compliant with this definition were included in the analysis.

DMOs were estimated across all locations combined and separately within indoor and outdoor locations. We selected specific WB thresholds to aggregate DMOs from based upon our research questions. For walking activity outcomes, we estimated the number of WBs undertaken per day to test whether the amount of walking changes with respect to contextual location. Walking-activity DMOs were estimated in accordance with Mobilise-D data aggregation guidelines (Koch et al., 2023). Gait-DMOs, were estimated from all WBs > 10 seconds, short WBs between 10 to 30 s and longer WBs > 30 likely to comprise steady-state gait and were hypothesized to mostly take place outdoors. This was done to explore how WB duration and location influence gait-control. DMOs of the gait domain cannot be estimated accurately and reliability from WBs < 10 s so they were removed.

### 2.4.1 Walking speed modes estimation

GMM was applied to walking speed to characterize the number of modes from the optimal number of clusters within its distribution. A GMM can be fitted with any fixed number of clusters, where each mode should correspond to a Gaussian distribution in the mixture and thus characterized by its mean parameter. GMMs were fitted using the “Mclust” function in the “Mclust” package (Scrucca et al., 2016) in R (R Foundation for statistical computing, V4.02, Austria). “Mclust” applies probabilistic, unsupervised machine learning algorithms, through a soft-clustering approach that identifies the probability that each data-point belongs to a specific mode. “Mclust” was first applied to estimate the number of modes *K* existing within the distribution of walking speed. The number of modes underlying the distribution of walking speed across all strides each participant took across the 7 days was determined by: (i) modeling each participant’s data using models assuming 1–9 modes; and then (ii) testing which of the models best fit the data using the Bayesian Information Criterion (BIC). BIC was implemented within the mclust framework, which compares Gaussian mixture models differing in the number of mixture components and covariance parameterizations, with model complexity explicitly penalized. The model with the lowest BIC value was selected as the optimal fit.

Mclust then applies an expectation maximization algorithm to fit the location and width of each walking speed mode to the individual’s distribution, where the width of each mode is given by the standard deviation of the corresponding Gaussian component in the mixture. Expectation-maximization algorithms are defined by an “*E-step*” which estimated the expected value for each walking-speed mode within the mixture and an “*M-step*” that optimized the parameters (mean and variance) of the distribution using maximum likelihood. Expectation-maximization algorithms assume modes that are randomly centered upon data points of walking speed and computed for each observation, the probability that it was generated by each walking speed mode of the model. The algorithm adjusted the mean and covariance parameters (μ, Σ) to maximize the fit of the mode given those parameters, this process is repeated until convergence is observed in the likelihood value. The number of walking speed modes were estimated separately from WBs > 10, between 10 and 30 and > 30 s.

### 2.5 Statistical analysis

All statistical analyses were completed in R. DMOs were summarized as continuous variables in the present analysis. Categorical variables were shown as counts and percentages, and continuous variables described as mean and standard deviation (SD), or median and 25th (P25) and 75th (P75) percentiles dependent upon normality. For inferential analyses, DMOs and clinical predictors were treated as continuous variables unless otherwise stated, while sex and cohort were treated as categorical variables. Continuous predictors were standardized prior to modeling. Model assumptions were assessed using standard diagnostic procedures, including inspection of residuals and assessment of multicollinearity.

#### 2.5.1 Comparison between indoor and outdoor locations

Comparisons between indoor and outdoor locations were made separately for both cohorts using generalized linear models (GLMs), adjusted for sex, age, and height. Separate models were run for PwP and controls to examine within-group effects and avoid potential interaction effects between cohort and environment that could obscure cohort-specific trends.

#### 2.5.2 Comparison between PD and control participants across indoor and outdoor locations

Comparisons between PwP and control participants were performed using GLMs that included both cohort and location as fixed effects, with an interaction term (Cohort × Location), adjusted for sex, age, and height. This allowed formal testing of whether the environment effect differed between cohorts. As several DMOs were examined, results are considered exploratory and no formal correction for multiple comparisons was applied. Instead, findings are interpreted with caution, focusing on effect sizes and the consistency of trends across outcomes.

#### 2.5.3 Characterization of walking speed modes

The optimal number of modes estimated within the distribution of walking speed were reported for each WB threshold and separately for PwP and control participants. This will be descriptively analyzed before comparing across WB thresholds and cohorts with Fisher’s exact testing.

To assess the effect of sample size on mode estimation, participants’ stride-level walking speed data were resampled to fixed stride counts prior to Gaussian Mixture Modeling (GMM). Resampling was performed in R using linear interpolation (approx function), applied separately to each participant’s daily stride data. For participants with fewer strides than the target count, stride data were up-sampled via interpolation to reach the required number. For participants with more strides than the target, the stride distribution was down-sampled to the specified count to ensure consistent input across participants. Fixed stride counts of 2,500, 5,000, 7,500, and 10,000 were selected to span the typical range of daily walking activity observed in the cohort and to examine how increasing stride count influences the stability and resolution of estimated walking speed modes. By systematically varying stride count and applying identical modeling procedures, this approach enabled evaluation of convergence and robustness of mode estimates while controlling for inter-individual differences in available stride data.

#### 2.5.4 Associations between walking speed modes and clinical information

Ordinal logistic regression was used to model the association between clinical characteristics and the ordinal outcome representing the number of walking speed modes across walking-bout duration thresholds. Models were specified using a proportional odds structure, in which the effects of predictors were assumed to be constant across cumulative outcome categories. Candidate predictors included MDS-UPDRS Parts II and III, levodopa equivalent daily dose (LEDD), number of daily medication intakes, freezing of gait questionnaire total score, Parkinson’s disease duration, late-life function and disability index (LLFDI), mass, and height. Backward elimination was applied to iteratively remove non-significant predictors (p > 0.05). Predictors retained consistently across duration thresholds were subsequently entered into a final multivariable model, with all predictors standardized and adjusted for age and sex. Model estimates were summarized using odds ratios with 95% confidence intervals, and model explanatory power was quantified using Nagelkerke’s R^2^.

## 3 Results

Thirty-nine PwP were included from the CiC study, and 19 controls from the TVS. In comparison to controls PwP were significantly taller, younger with a larger proportion of males (62% vs. 47%). Motor scores in the PwP were low (median MDS-UPDRS = 10 points) (Table 1).

**TABLE 1:** Demographic and clinical data for people with PD (PwP) and control participants.

Group	Control	PwP	*P*-values
**n**	17	39	–
Age (y)*	70, (67, 74)	64 (57, 71.5)	0.010
**Sex (% male, n male/n female)**	47% (8/9)	62%, (24/15)	0.233
Height (meters)*	1.66 (1.56, 1.75)	1.7 (1.6, 1.76)	0.292
**Body mass (kg)**	75.8, (62, 82)	73.6 (67.9, 82.3)	0.955
**Real-world walking speed (m/s)**	0.90 (0.79, 1.02)	0.82 (0.78, 0.91)	0.479
**PD duration (years)**	–	3.92 (2.41, 6.37)	–
**LLFDI (points)**	–	135 (122.5, 145.5)	–
**LEDD (mmhg)**	–	475 (350, 750)	–
**MDS-UPDRS II (points)**	–	10 (6, 14)	–
**MDS-UPDRS III (points)**	–	24 (10.5, 32)	–
**Hoehn and Yahr stage**			
**I, n (%)**	–	1 (2%)	–
**II, n (%)**	–	37 (94%)	–
**III, n (%)**	–	1 (2%)	–

Data presented as median (25th and 75th percentiles). LLFDI, The Late Life Function and Disability Index; LEDD, Levodopa equivalent daily dosage; MDS-UPDRS III and II, Movement Disorder Society – Unified Parkinson’s Disease Rating Scale – Part III. “*” denotes significantly different between PD and Controls.

### 3.1 Characterization of indoor and outdoor walking

Of the 52 PwP included in the study, 39 (75%) had usable contextual data. Participants without usable contextual data was due to technical issues with the devices (Supplementary Table 1). These participants contributed a median of 7 days of contextual monitoring (range: 2–7 days). Across these days, a total of 113,725 WBs were detected, with a median of 2,017 WBs per participant (range: 970–4,850). Of these, 77,314 WBs were classified as valid for contextual analysis, corresponding to 67% of all detected WBs. The median number of valid WBs per participant was 1,757 (range: 592–4,079).

Among the 19 older adult control participants, 17 (89%) had contextual data available. They also contributed a median of 7 days of data (range: 1–7 days). In total, 38,372 WBs were detected, with a median of 1,984 per participant (range: 670 to 3,482). Of these, 30,895 WBs (80%) were deemed valid, with a median of 1,826 valid WBs per participant (range: 355–3,465).

### 3.2 Comparison of indoor and outdoor walking characteristics

**FIGURE 1:**
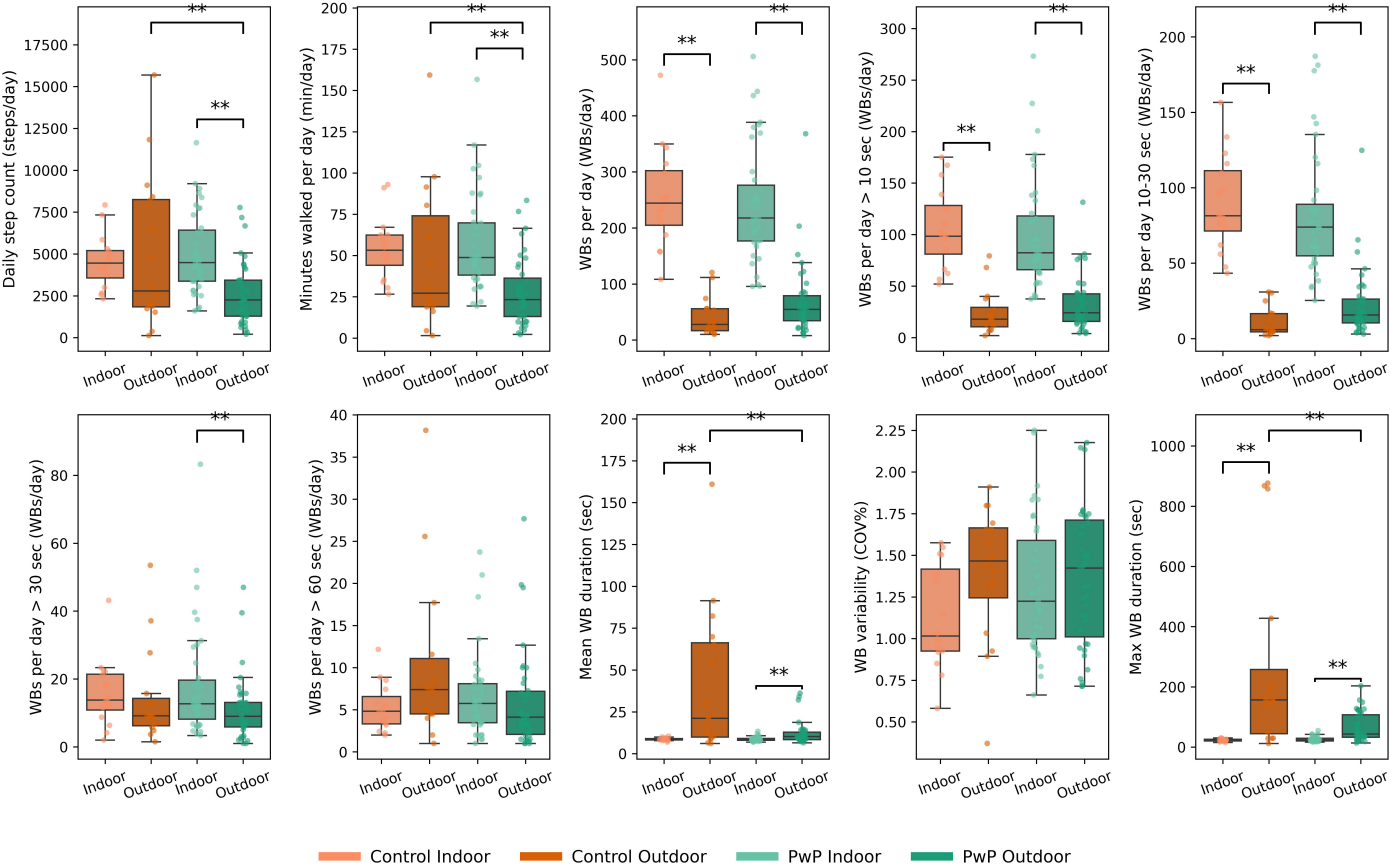
Boxplots displaying median walking digital mobility outcomes (DMOs) measured across indoor and outdoor locations for controls and people with PD (PwP). Datapoints represent individual participants. Indices and “**” indicates significant difference (GLM *P* = < 0.01).

PwP walked significantly more indoors than outdoors; for example, they took 4,480 steps per day, 48.8 min of walking, and 217 walking bouts per day indoors, compared with 2,253 steps per day, 23.3 min of walking, and 54 walking bouts per day outdoors. They also undertook significant more short WBs (between 10 and 30 s) indoors, although there was no significant difference between indoor and outdoor WBs for durations > 30 s or > 60 s. PwP had significantly longer mean and maximum daily WB duration outdoors (mean WB duration: 10.2 s vs. 8.4 s; maximum WB duration: 43.2 vs. 24.2 sec) (Figure 1; Supplementary Table 2; Supplementary Figure 1).

For control participants, whilst they trended toward larger amounts of indoor than outdoor walking (4,449 vs. 2,788 steps per day; 53.2 vs. 27.2 min walking per day), this was not significantly different. They did undertake significantly more indoor than outdoor WBs > 10 s and short WBs between 10 and 30 s with no significant differences in WBs > 30 or > 60 s (*p* > 0.05). Similar to PwP both mean and maximum WB duration were significantly longer outdoors in comparison to indoors (mean WB duration (21.1 vs. 8.6 s, *p* = 0.006) and maximum WB (22.3 vs. 156.9 s, *p* = 0.005) (Figure 1; Supplementary Table 2; Supplementary Figure 1).

### 3.3 Comparison of indoor and outdoor gait characteristics

**FIGURE 2:**
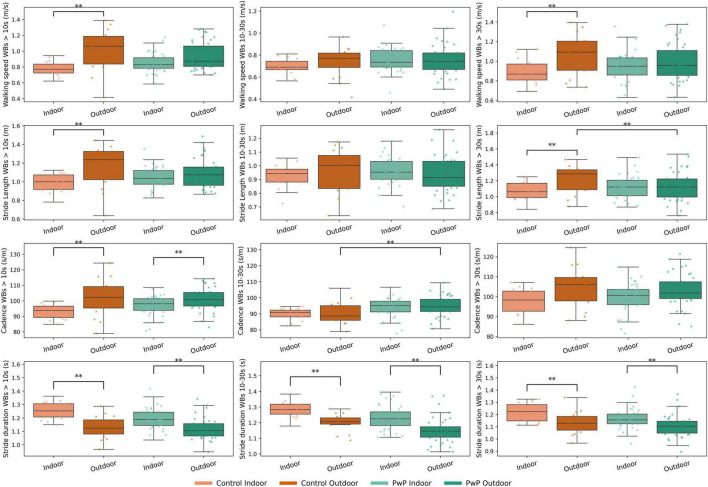
Boxplots displaying gait digital mobility outcomes (DMOs) measured across indoor and outdoor locations for People with Parkinson’s (PwP) and controls. Datapoints represent individual participants. Indices and “**” indicates significant difference (GLM *P* = < 0.01).

PwP walked at a similar pace in both indoor and outdoor locations, with no significant differences between walking speed and stride length (*P* > 0.05). In the rhythm domain, cadence was significantly lower and stride duration significantly higher indoors compared to outdoors across all WBs > 10 s, and stride duration was significantly lower indoors compared to outdoors across each WB duration (P < 0.01) (Figure 2) (Supplementary Table 4; Supplementary Figure 3).

In contrast to PwP, controls showed location-specific differences in pace, with walking speed and stride length being significantly faster and longer, respectively, across all WBs > 10 s and WBs > 30 s. Similar to PwP controls also demonstrated a significantly longer stride duration indoors compared to outdoors across each WB threshold (Figure 2; Supplementary Table 4; Supplementary Figure 3).

### 3.4 Comparison between PD and control participants across indoor and outdoor locations

Across all location data combined, PwP undertook significantly less steps than controls walked (6,607 vs. 9,309 steps per day, *P* = 0.001) and walked for less time; (73.4 vs. 98.2 min per day, *P* = 0.003). No differences were observed for DMOs of the pattern domain.

No significant differences in indoor walking were observed between cohorts across walking and gait DMOs. Outdoors, however, PwP walked significantly less than controls (2,235 vs. 2,788 steps per day, *p* = 0.003; 23.3 vs. 27.7 min per day, *p* = 0.009) with shorter WBs (mean WB duration: 43.2 vs. 156.9 s, *p* = 0.001; maximum WB duration: 8.6 vs. 21.1 s, *p* = 0.001). These differences may be driven by an outlier in the OA cohort (Figure 1). Outdoors, PwP also exhibited a significantly higher cadence than controls during short WBs (94.9 vs. 90.3 steps/min, *p* = 0.009) (Supplementary Tables 2–5; Supplementary Figures 2, 4).

### 3.5 Characterization of walking speed modes

**FIGURE 3:**
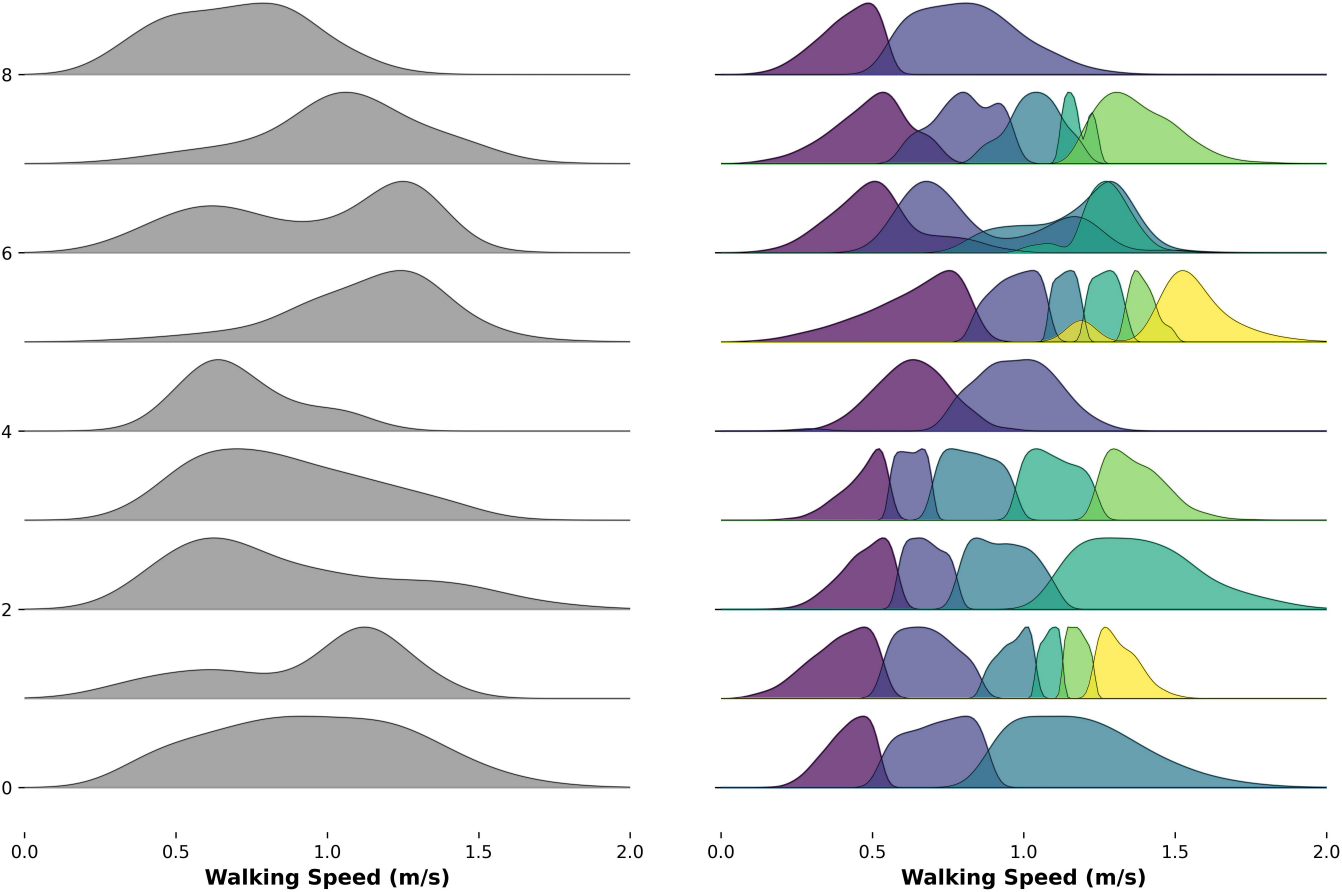
Ridged density plot showing selected examples of the number of modes identified within the raw distribution of real-world walking speed for people with PD and control participants.

Examples of the different number of walking speed modes are visualized in Figure 3.

Across each WB duration threshold, the largest proportion of PwP and control participants were best characterized by three walking speed modes (Table 2). No significant difference between cohorts or WB thresholds was observed (Fisher’s exact text *P* > 0.05).

**TABLE 2:** Distribution of walking speed modes across walking bout (WB) durations.

Walking speed mode number	1	2	3	4	5
**Parkinson’s (*n* = 52)**					
WBs > 10 s (n, %)	0 (0%)	3 (5%)	**30 (57%)**	14 (26%)	5 (9%)
WBs 10–30 s (n, %)	0 (0%)	14 (26%)	**37 (71%)**	1 (1%)	0 (0%)
WBs > 30 s (n, %)	0 (0%)	8 (15%)	**32 (61%)**	11 (21%)	1 (1%)
**Older adults (*n* = 19)**					
WBs > 10 se (n, %)	0 (0%)	4 (21%)	**8 (42%)**	6 (13%)	1 (5%)
WBs 10–30 s (n, %)	1 (5%)	**9 (47%)**	**9 (47%)**	0 (0%)	0 (0%)
WBs > 30 s (n, %)	0 (0%)	3 (15%)	**9 (52%)**	7 (36%)	0 (0%)

The number and proportion of walking speed modes are presented for each cohort across each different mode type that was identified by mclust. Highlighted in bold denotes the number of walking modes identified in the largest proportion of participants across each WB threshold.

When re-sampling to a fixed number of strides, slight changes were observed in the number of walking speed modes estimated for the larger stride samples (7,500 and 10,000), where individuals were predominantly characterized by four and five strides, respectively. The pattern was consistent across both cohorts (Supplementary Table 6).

### 3.6 Associations between clinical variables and the number of walking speed modes

The only associations between the number of walking speed modes and clinical variables were found at WBs > 30 s. A 100 mg increase in LEDD was associated with higher odds of having a greater number of walking speed modes (OR = 1.24, 95% CI [1.009, 1.562]). Similarly, a 5-point increase in MDS-UPDRS III was associated with higher odds of more walking speed modes (OR = 1.037, 95% CI [1.009, 1.070]) Partial effects of both LEDD and MDS-UPDRS III upon the predicted probability of a higher number of walking speed modes can be found in Supplementary Figures 5, 6.

## 4 Discussion

The primary aim of this study was to explore the utility of indoor and outdoor location information in its ability to contextualize real-world mobility in PwP and control participants. Despite data loss, this study demonstrated feasibility of smartphones to capture indoor outdoor contextual factors which showed promise in providing novel insight into both walking behavior and functional gait DMOs. Most notably, both cohorts engaged in more walking indoors than outdoors, with shorter WBs occurring predominantly indoors; only the control participants adapted their walking speed differently between locations. Group differences emerged only in DMOs estimated from outdoor walking, where people with PD took significantly fewer steps and accumulated fewer minutes walked per day compared to controls. This study also examined a novel approach to characterizing walking behavior by identifying walking speed modes, which was similar across cohorts with individual variation. Walking speed modes showed moderate associations with motor severity, though further research is needed to establish their potential as a clinically meaningful feature in PD.

This study is the first to combine GPS-derived indoor outdoor contextual data with wearable-derived DMOs. 82% of participants had 7-days of usable contextual data demonstrating feasibility and aligning with published recommendations (Buekers et al., 2024). PwP had a lower proportion of WBs with valid contextual data (PD: 65% vs. controls: 80%). The specific reasons for invalid data were not captured but may relate to common GPS limitations rather than PD. Determining location from GPS is subject to noise, caused by occlusion (Kerr et al., 2011) and reflection that is particularly an issue is urban environments. In addition, the approach relies on mapping data, which suffers from omissions and update delays. Data loss could also be due to practical issues such as smartphones running out of battery if not placed on the Bluetooth charger. In the CiC cohort, Debelle et al. (2023) reported that selected participants found the smartphone a “nuisance,” being “bulky,” or “too big” to carry, finding limited benefit from the device. Future studies may benefit from smaller GPS-enabled devices and from capturing detailed metadata on participant’s living environments (urban vs. rural), which could help clarify sources of data loss, whether it’s due to technical issues or the presence of PD.

Contextual factors showed promise in improving our interpretation of real-world walking behaviors. Both cohorts undertook significantly more walking indoors in keeping with our hypothesis, based upon previous self-reported surveys that have determined humans spend 90% of their time indoors (Diffey, 2011). Additionally, short WBs predominantly took place indoors suggesting that activity here likely represents walking in the context of everyday activities completed in the household or work setting. Additionally, the mean daily WB duration was significantly longer outdoors than indoors for both cohorts, partially supporting our hypothesis that most long WBs would occur outdoors. However, both cohorts performed more WBs > 30 s indoors, reaching statistical significance only for PwP. For WBs > 60 s, only controls showed a non-significant trend toward more outdoor bouts. The overall number of long WBs was low, and the effect sizes at this duration were small (Supplementary Figure 1), suggesting limited statistical power to detect differences. These results may also reflect GPS limitations in distinguishing truly indoor from near-indoor locations (e.g., gardens) or transitions between indoors and outdoors, which GPS cannot reliably capture. Both cohorts undertook a similar amount of indoor walking. However, outdoors PwP undertook significantly less steps, minutes walked per day with a shorter mean and maximum WB duration. This may reflect environmental factors (e.g., controls living in more walkable areas with green spaces) or lifestyle factors such as commuting or greater physical activity. Conversely, people with PD may leave the house only when ‘ON’ medication or accompanied, with fatigue, motor severity, and wearing-off effects limiting outdoor activity. This highlights the complex interplay of factors shaping real-world mobility and the importance of contextual information for more precise clinical insight. As physical activity is neuroprotective in PD (Speelman et al., 2011), maintaining outdoor mobility is especially important.

Contextual factors also showed potential to improve interpretation of gait DMOs. At the gait-level adaptation between locations in walking speed and stride length were only observed for controls. It was expected that walking speed would be slower in more confined and cluttered indoor spaces (Wettstein et al., 2013; Wettstein et al., 2015), requiring a different adaption to outdoors. No location differences in walking speed among PwP may reflect PD-related factors such as impaired muscle contraction, rigidity, and postural instability affecting adaptability (Mirelman et al., 2019), or unmeasured environmental and lifestyle factors, such as more walkable environments that support longer steady-state gait or purposeful walking (e.g., commuting). When comparing cohorts, PwP trended to walk slower outdoors in comparison to controls across all WB categories, though differences were not significant. Perhaps lack of significance was driven by different group numbers and low sample sizes (Supplementary Figure 4). Stride length was significantly shorter during long WBs (>30 s), suggesting that PwP may be unable to generate the same spatial propulsion as OAs during longer, more optimal walking. PwP also showed significantly higher cadence during short outdoor WBs, indicating a more shuffling gait during intermittent outdoor walking. Cadence is not as widely quantified in studies as step or stride duration (Kirk et al., 2025), however this finding is consistent with recent literature (Yarnall et al., 2025). Stride duration tended to be lower in PwP across WBs, but differences were not significant. Overall, contextual factors revealed no strong group differences, likely influenced by unequal group sizes and mild motor severity in PwP. Larger, more evenly matched cohorts are needed to fully explore these differences across contexts.

Additionally, this study explored the utility of an unsupervised machine-learning technique to estimate the number of walking speed modes. The largest proportion of PwP and control participants had three walking speed modes across each duration threshold. Within a single WB, walking speed was expected to demonstrate three distinct phases: acceleration, steady-state, and deceleration, with additional variability to adapt to real-world demands. These findings support this hypothesis and expand upon existing studies which typically fit a GMM of two modes (faster and slower) (Atrsaei et al., 2021; Brodie et al., 2017; Van Ancum et al., 2019). More recently, Baroudi et al. (2022) employed a multi-level modeling approach and identified an optimal number of five walking speed modes across all individuals. However, comparisons are limited as their population consisted of young athletes who were assessed across 14 days. The limited differences in walking speed modes observed here may reflect the relatively mild motor severity of the PwP, as most participants were unlikely to experience pronounced medication fluctuations or FOG that typically drive greater variability (Mirelman et al., 2019; Packer et al., 2025). Furthermore, walking speed modes remained consistent across WB duration thresholds, suggesting stable gait modulation patterns across real-world activities. Although re-sampling participants to include larger step counts (7,500 and 10,000) increased the number of detected modes, this likely reflects the influence of larger sample sizes improving model resolution rather than true behavioral differences (Burnham and Anderson, 2004). Therefore, the relationship between data volume and estimated modes should be interpreted cautiously, as it may not indicate genuine changes in walking adaptability.

Clinical predictors only emerged with a greater number of walking speed modes estimated from walking bouts > 30 s, specifically higher medication dosage (LEDD) and greater motor severity (MDS-UPDRS III) (Supplementary Figures 5, 6). Walking speed in longer bouts reflects optimal steady-state gait, similar to laboratory walking, and is characterized by greater consistency and lower variability (Corrà et al., 2021). In contrast, higher LEDD often indicates longer disease duration and more advanced symptoms, both of which can fluctuate within and between days. Similarly, higher MDS-UPDRS III scores reflect more severe motor impairments, which can cause inconsistency in ability to regulate walking speed and stride length within a single WB. Together, these factors increase variability in walking speed during prolonged bouts, resulting in larger number of walking sped modes. Prolonged bouts provide sufficient temporal resolution to capture these fluctuations, whereas shorter bouts are typically too brief to reveal multiple modes. Previous studies have demonstrated ON–OFF medication differences in walking speed, with one (Corrà et al., 2021) reporting greater impairment in short, rather than long WBs; however, that analysis relied on singular mean and maximum values. Atrsaei et al. (2021) employed a bimodal approach and found correlations between LEDD and walking speed extracted from the highest mode in the distribution, which alongside the current approach, suggests a possible link between walking speed modes and medication. Nevertheless, caution is advised, as participants in the CiC study were in the early stages of motor symptom progression, and some may not have experienced medication-related fluctuations. Replication in a larger, more advanced PD cohort is needed before drawing stronger conclusions.

For limitations, this study had relatively low numbers overall, particularly for control participants which may have affected statistical comparisons. Additionally, PwP were assessed in a singular location, had mild motor severity and limited to H&Y stage 1–3, which limits generalizability of results. Effect sizes of specific DMOs were close to “0” suggesting statistical analysis was underpowered (Supplementary Figures 1–4). Future studies are needed with larger more diverse cohorts. Despite the exciting potential of GPS technology, a comprehensive technical validation considering algorithms, GPS receiver and device wear-location (Kerr et al., 2011; Schipperijn et al., 2014) is required. Recent approaches have adopted machine-learning and deep learning to estimate context based solely upon magnetometer data demonstrating high accuracy in comparison to GPS measurements (Marcianò et al., 2025). Additional DMOs related to turns captured indoors would provide novel insight into how PD affects real-world mobility (Mancini et al., 2018). Walking speed modes was not extended to other DMOs, as our initial aim was to evaluate the feasibility of the approach on a single, representative outcome. Walking speed was selected as it is derived from multiple underlying DMOs and is widely recognized as a global indicator of motor performance (Kirk et al., 2024b; Middleton et al., 2015). Walking speed modes must be replicated in larger and more diverse cohorts of PwP and is complex in its interpretation. Thus, a more comprehensive explanatory analysis is required to establish clinical meaningfulness including information related to falls risk, muscle force (Pelicioni et al., 2021), balance measures (Mancini et al., 2011; Mancini et al., 2012) and psychological factors such as anxiety and fear.

Integrating GPS-derived contextual information with DMOs offers a promising approach to enhance understanding of the factors influencing real-world mobility in PD and to explore how mobility is modulated across indoor and outdoor environments. Further technical validation is needed to refine the collection and analysis of GPS data and to evaluate its applicability in larger, more heterogeneous PD cohorts. Emerging features such as walking speed modes show promise for capturing the fluctuating and adaptive nature of mobility in PD, but their clinical relevance and robustness require confirmation through clinical validation in independent datasets.

## Data Availability

The raw data supporting the conclusions of this article will be made available by the authors, without undue reservation.
