# Supplementary material for: Toward an understanding of real-world mobility in Parkinson’s: insights from enhanced contextualisation using GPS-derived location and data-driven modeling of walking speed
Source: Front Aging Neurosci. 2026 Feb 20;18:1746429. doi: 10.3389/fnagi.2026.1746429 (PMC12963274; doi:10.3389/fnagi.2026.1746429)
Supplement: Supplementary file 1 [file Data_Sheet_1.docx]

Supplementary Material

# Supplementary Tables

**Supplementary table 1.** Demographic and clinical data for PwP without valid contextual data. Data presented as median (25^th^ and 75^th^ percentiles) or mean (standard deviation).

| Group | PwP excluded from analysis | PwP included analysis |
| --- | --- | --- |
| n | 13 | 39 |
| Age (y) | 68 (61, 73) | 64 (57, 71.5) |
| Sex (%Male, n Male / n Female) | 11, 2 (84%) | 24, 1 (96%) |
| Height (metres) | 1.77 (1.7, 1.84) | 1.7 (1.6, 1.76) |
| Body Mass (kg) | 87 (78.2, 95) | 73.6 (67.9, 82.3) |
| Real-world walking speed (m/s) | 0.85 (0.76, 0.92) | 0.82 (0.78, 0.91) |
| PD duration (years) | 4.33 (2.42, 5.52) | 3.92 (2.41, 6.37) |
| LLFDI (points) | 134 (122, 144) | 135 (122.5, 145.5) |
| LEDD (mmhg) | 575 (400, 907) | 475 (350, 750) |
| MDS-UPDRS II (points) | 8 (4, 15) |  |
| MDS-UPDRS III (points) | 8 (5,14) | 24 (10.5, 32) |
| Hoehn and Yahr Stage |  |  |
| I, n (%) | 1 (7%) | 1 (2%) |
| II, n (%) | 11 (84%) | 37 (94%) |
| III, n (%) | 1 (7%) | 1 (2%) |

MDS-UPDRS III = Movement Disorder Society – Unified Parkinson’s Disease Rating Scale – Part III.

**Supplementary table 2**. Summary of walking outcomes estimated from all, indoor and outdoor locations for Parkinson’s (PD) and older adults (Controls). Data presented as median (25th percentile, 75th percentile)

| DMO | PD All | PD Indoor | PD outdoor | OA All | OA Indoor | OA outdoor |
| --- | --- | --- | --- | --- | --- | --- |
| Amount | | | | | | |
| Steps per day (number) | 6607.1  (5411.2, 9247.2) | 4480.4  (3371.1, 6418.0) | 2253.5  (1280.6, 3437.1) | 9309.7  (7098.6, 11610.8) | 4449.4  (3568.6, 5203.3) | 2788.1  (1839.9, 8245.8) |
| Daily walking duration (minutes) | 73.4  (59.8, 103.4) | 48.8  (38.1, 69.8) | 23.3  (13.1, 36.3) | 98.2  (85.9, 127.2) | 53.2  (44.1, 62.4) | 27.2  (19.0, 74.1) |
| Pattern | | | | | | |
| Walking bouts per day (number) | 288.2  (236.1, 324.9) | 217.8  (176.7, 276.1) | 54.7  (34.4, 78.9) | 288.7  (246.1, 332.5) | 244.2  (204.8, 302.0) | 28.0  (16.6, 55.8) |
| Walking bouts per day > 10 seconds (number) | 117.4  (87.8, 155.3) | 82.3  (65.9, 118.0) | 24.1  (15.7, 42.5) | 129.4  (115.9, 160.4) | 98.4  (81.0, 128.3) | 17.9  (10.5, 29.3) |
| Walking bouts per day 10-30 seconds (number) | 94.6  (71.4, 116.2) | 73.9  (54.9, 89.1) | 15.7  (10.3, 26.1) | 93.6  (76.8, 121.9) | 81.4  (71.3, 111.3) | 5.9  (4.5, 16.6) |
| Walking bouts per day > 30 seconds (number) | 20.9  (12.6, 36.8) | 12.7  (8.2, 19.6) | 9.0  (5.9, 13.1) | 25.9  (19.1, 38.6) | 13.8  (10.9, 21.4) | 9.2  (6.2, 14.3) |
| Walking bouts per day > 60 seconds (number) | 9.3  (4.8, 12.7) | 5.8  (3.5, 8.1) | 4.1  (2.1, 7.2) | 10.8  (8.2, 16.9) | 4.8  (3.3, 6.6) | 7.4  (4.5, 11.1) |
| Walking bout duration (seconds) | 8.6  (8.2, 9.3) | 8.4  (8.0, 9.1) | 10.2  (8.4, 12.8) | 9.1  (8.7, 9.5) | 8.6  (8.2, 9.1) | 21.1  (9.9, 66.3) |
| Maximum walking bout duration (Seconds) | 27.2  (21.7, 32.1) | 24.2  (20.6, 29.4) | 43.2  (32.5, 107.2) | 27.8  (23.8, 42.0) | 22.3  (20.5, 25.8) | 156.9  (44.4, 257.6) |
| Walking bout variability (COV %) | 1.5  (1.1, 1.7) | 1.2  (1.0, 1.6) | 1.4  (1.0, 1.7) | 1.9  (1.5, 3.1) | 1.0  (0.9, 1.4) | 1.5  (1.2, 1.7) |

**Supplementary table 3.** P values from the Generalised linear model compared walking outcomes between indoor and outdoor locations and across. All models were adjusted for sex and age.

| DMO | PD indoor vs Outdoor | HA indoor vs outdoor | PD vs HA All locations | PD vs HA ‘indoor’ locations | PD vs HA outdoor locations |
| --- | --- | --- | --- | --- | --- |
| Amount | | | | | |
| Steps per day (number) | 0.000 | 0.724 | 0.001 | 0.378 | 0.003 |
| Daily walking duration (minutes) | 0.000 | 0.564 | 0.003 | 0.205 | 0.009 |
| Pattern | | | | | |
| Walking bouts per day (number) | 0.000 | 0.000 | 0.535 | 0.163 | 0.430 |
| Walking bouts per day > 10 seconds (number) | 0.000 | 0.000 | 0.253 | 0.049 | 0.758 |
| Walking bouts per day 10-30 seconds (number) | 0.000 | 0.000 | 0.458 | 0.050 | 0.250 |
| Walking bouts per day > 30 seconds (number) | 0.006 | 0.742 | 0.074 | 0.221 | 0.177 |
| Walking bouts per day > 60 seconds (number) | 0.493 | 0.070 | 0.022 | 0.913 | 0.048 |
| Mean walking bout duration (seconds) | 0.003 | 0.006 | 0.188 | 0.124 | 0.001 |
| Maximum walking bout duration (Seconds) | 0.000 | 0.005 | 0.069 | 0.557 | 0.001 |
| Walking bout variability (COV %) | 0.560 | 0.046 | 0.019 | 0.183 | 0.321 |

Supplementary table 4*.* Summary of gait outcomes estimated from all, indoor and outdoor locations for Parkinson’s (PD) and Controls. Data presented as mean (SD)

| DMO | PD All | PD Indoor | PD outdoor | Controls All | Controls Indoor | Controls outdoor |
| --- | --- | --- | --- | --- | --- | --- |
| Pace | | |  |  |  |  |
| Walking speed in all (> 10s) WBs (m/s) | 0.88 (0.14) | 0.86 (0.12) | 0.93 (0.17) | 0.95 (0.19) | 0.77 (0.09) | 1.03 (0.27) |
| Walking speed in shorter (10-30s) WBs (m/s) | 0.75 (0.10) | 0.76 (0.11) | 0.76 (0.14) | 0.71 (0.07) | 0.69 (0.07) | 0.73 (0.13) |
| Walking speed in longer (>30s) WBs (m/s) | 0.96 (0.15) | 0.94 (0.15) | 0.98 (0.19) | 1.03 (0.20) | 0.88 (0.12) | 1.09 (0.22) |
| Stride length in all (> 10s) WBs (m/s) | 1.06 (0.12) | 1.04 (0.11) | 1.09 (0.16) | 1.12 (0.15) | 0.99 (0.09) | 1.18 (0.22) |
| Stride length in shorter (10-30s) WB (m) | 0.95 (0.10) | 0.96 (0.09) | 0.94 (0.13) | 0.94 (0.09) | 0.92 (0.08) | 0.96 (0.15) |
| Stride length in longer (>30s) WB (m) | 1.12 (0.14) | 1.12 (0.14) | 1.13 (0.18) | 1.19 (0.15) | 1.07 (0.11) | 1.23 (0.18) |
| Rhythm | | |  |  |  |  |
| Cadence in all (> 10s) WBs (steps/min) | 98.56 (7.17) | 97.05 (6.35) | 100.97 (7.09) | 99.10 (9.98) | 92.37 (4.95) | 102.93 (12.52) |
| Cadence in shorter (10-30s) WBs (steps/min) | 93.91 (6.17) | 93.76 (6.25) | 94.95 (6.27) | 89.28 (3.71) | 89.19 (3.83) | 90.33 (6.66) |
| Cadence in longer (>30s) WBs (steps/min) | 101.10 (7.92) | 99.58 (7.36) | 103.21 (8.14) | 102.04 (10.67) | 96.78 (6.22) | 105.20 (10.92) |
| Stride duration in all (> 10s) WBs (s) | 1.17 (0.08) | 1.20 (0.08) | 1.12 (0.08) | 1.21 (0.09) | 1.26 (0.06) | 1.13 (0.09) |
| Stride duration in shorter WBs (10-30s) WBs | 1.22 (0.07) | 1.23 (0.08) | 1.15 (0.08) | 1.28 (0.05) | 1.29 (0.05) | 1.20 (0.06) |
| Stride duration in longer (>30s) WBs (s) | 1.15 (0.09) | 1.17 (0.08) | 1.10 (0.09) | 1.18 (0.10) | 1.22 (0.08) | 1.13 (0.09) |

**Supplementary table 5.** P values from the Generalised linear model compared gait outcomes between indoor and outdoor locations and across. All models were adjusted for sex and age.

| DMO | PD indoor vs Outdoor | HA indoor vs outdoor | PD vs HA All locations | PD vs HA ‘indoor’ locations | PD vs HA outdoor locations |
| --- | --- | --- | --- | --- | --- |
| Pace | | |  |  |  |
| Walking speed in all (> 10s) WBs (m/s) | 0.014 | 0.003 | 0.046 | 0.462 | 0.056 |
| Walking speed in shorter (10-30s) WBs (m/s) | 0.931 | 0.330 | 0.548 | 0.427 | 0.774 |
| Walking speed in longer (>30s) WBs (m/s) | 0.184 | 0.007 | 0.031 | 0.559 | 0.028 |
| Stride length in all (> 10s) WBs (m/s) | 0.121 | 0.005 | 0.008 | 0.960 | 0.029 |
| Stride length in shorter (10-30s) WB (m) | 0.327 | 0.376 | 0.506 | 0.896 | 0.422 |
| Stride length in longer (>30s) WB (m) | 0.713 | 0.013 | 0.006 | 0.324 | 0.011 |
| Rhythm | | | | | |
| Cadence in all (> 10s) WBs (steps/min) | 0.006 | 0.013 | 0.939 | 0.067 | 0.652 |
| Cadence in shorter (10-30s) WBs (steps/min) | 0.374 | 0.693 | 0.012 | 0.039 | 0.009 |
| Cadence in longer (>30s) WBs (steps/min) | 0.028 | 0.033 | 0.775 | 0.647 | 0.660 |
| Stride duration in all (> 10s) WBs (s) | 0.000 | 0.000 | 0.414 | 0.041 | 0.932 |
| Stride duration in shorter WBs (10-30s) WBs | 0.000 | 0.000 | s0.009 | 0.043 | 0.052 |
| Stride duration in longer (>30s) WBs (s) | 0.001 | 0.011 | 0.535 | 0.178 | 0.691 |

Significant differences (p < 0.01) highlighted in bold

**Supplementary table 6.** Distribution of walking speed modes estimated from data resampled to a fixed number of strides. The number and proportion of walking speed modes are presented for each cohort across each different mode type that was identified by mclust.

| Parkinson’s (n = 52) | | | | | |
| --- | --- | --- | --- | --- | --- |
| Walking speed mode number | 2 | 3 | 4 | 5 | 6 |
| 2500 strides | 11 (21%) | 32 (61%) | 9 (17%) | 0 (0%) | 0 (0%) |
| 5000 strides | 1 (1%) | 29 (55%) | 20 (38%) | 2 (3%) | 0 (0%) |
| 7500 strides | 0 (0%) | 14 (26%) | 25 (48%) | 12 (23%) | 1 (1%) |
| 10000 strides | 0 (0%) | 7 (13%) | 22 (42%) | 16 (30%) | 7 (13%) |
| Older adults (n = 17) | | | | | |
| Walking speed mode number | 2 | 3 | 4 | 5 | 6 |
| 2500 strides | 5 (29%) | 11 (64%) | 1 (6%) | 0 (0%) | 0 (0%) |
| 5000 strides | 2 (11%) | 8 (53%) | 6 (40%) | 1 (6%) | 0 (0%) |
| 7500 strides | 2 (11%) | 5 (29%) | 8 (47%) | 2 (11%) | 0 (0%) |
| 10000 strides | 1 (6%) | 0 (0%) | 4 (23%) | 8 (47%) | 4 (23%) |

## Supplementary Figures

*
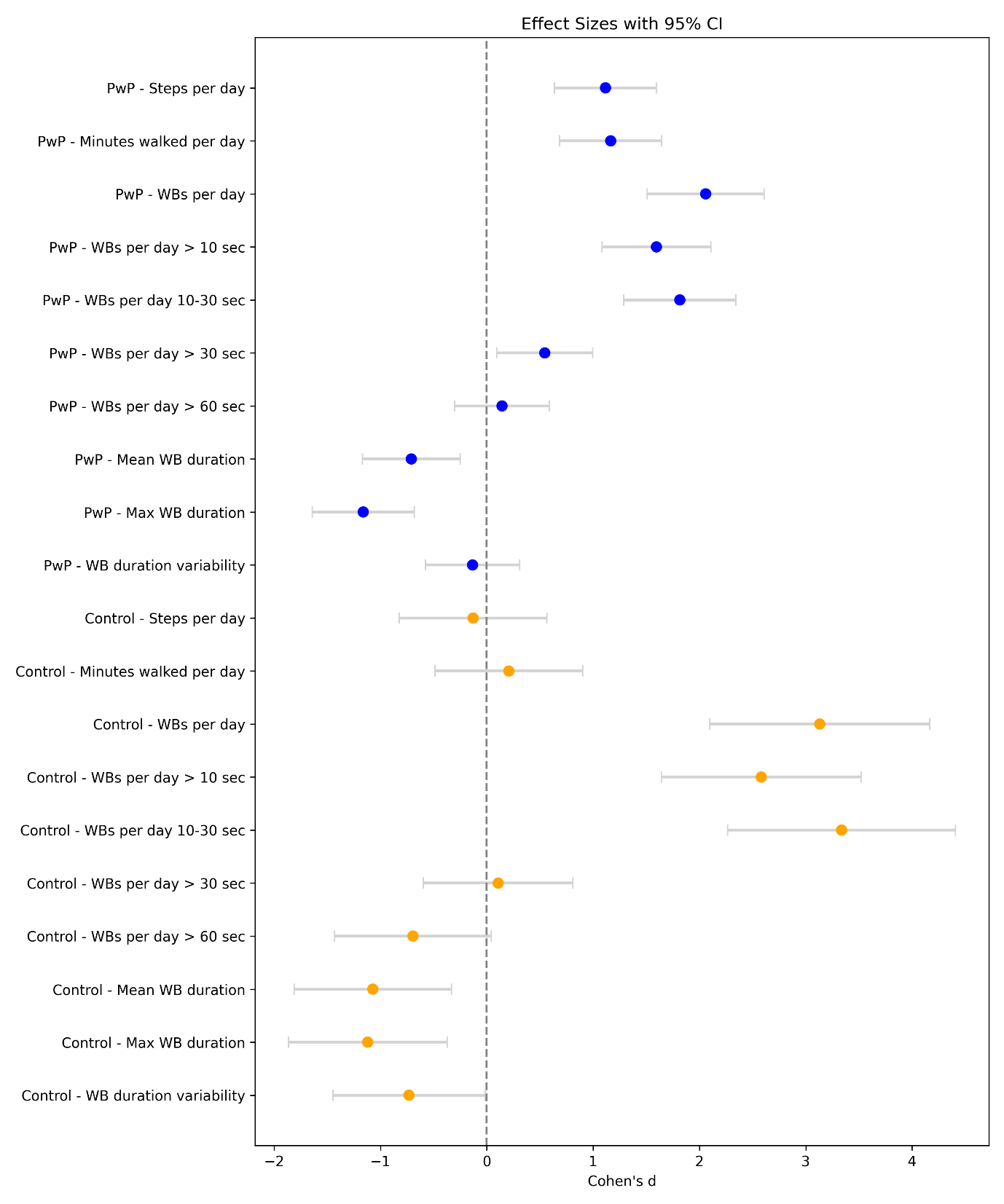
***Supplementary figure 1.** Effect sizes (Cohen’s d) for walking-related digital mobility outcomes compared between indoor and outdoor locations within each cohort. Points represent Cohen’s d values for each variable, with horizontal bars showing 95% confidence intervals. Data are grouped by cohort (People with Parkinson’s (PwP) and controls) and color-coded accordingly.


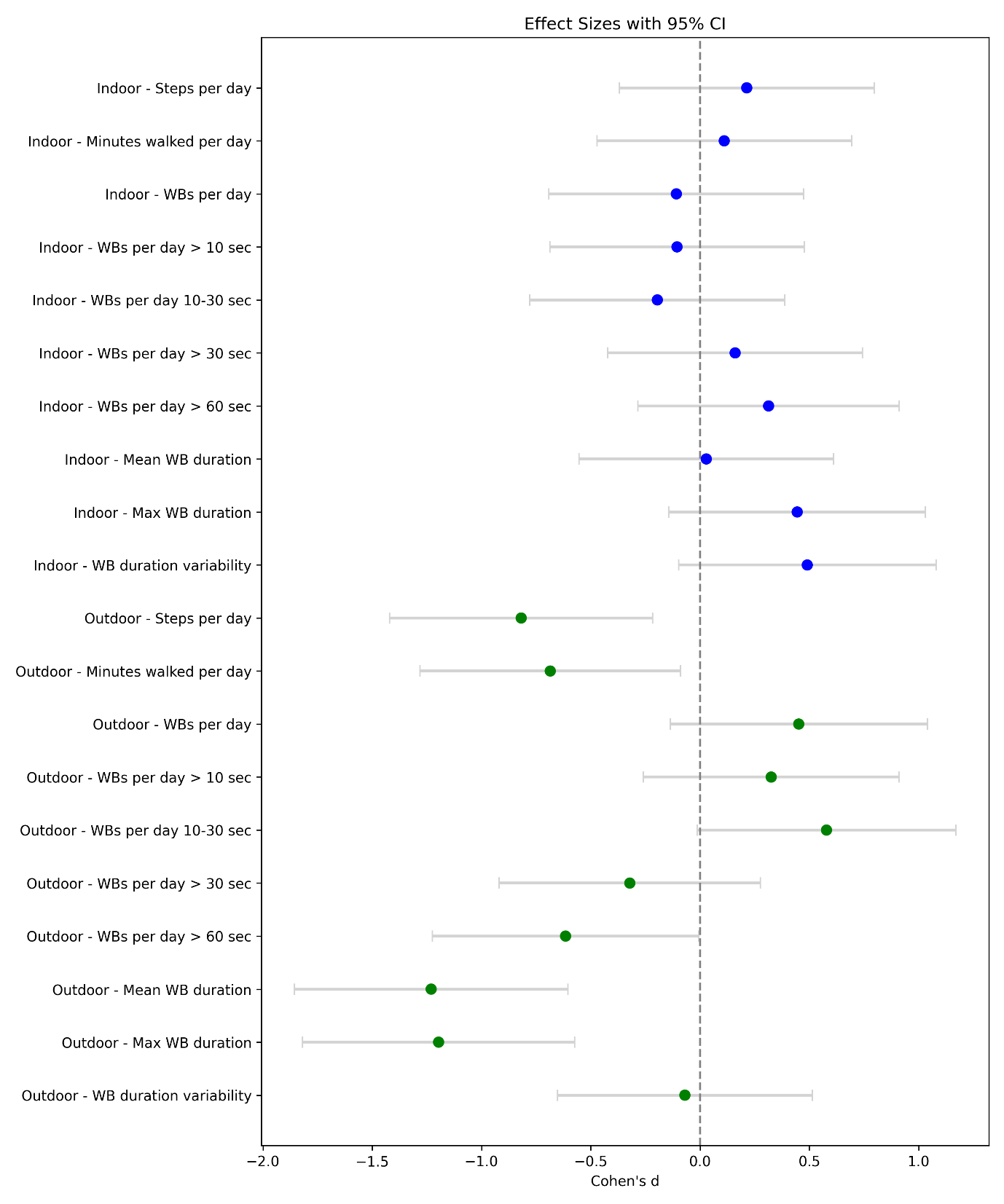
**Supplementary figure 2.** Effect sizes (Cohen’s d) for walking-related digital mobility outcomes compared between each cohort across indoor and outdoor locations. Points represent Cohen’s d values for each variable, with horizontal bars showing 95% confidence intervals. Data are grouped by location and color-coded accordingly.


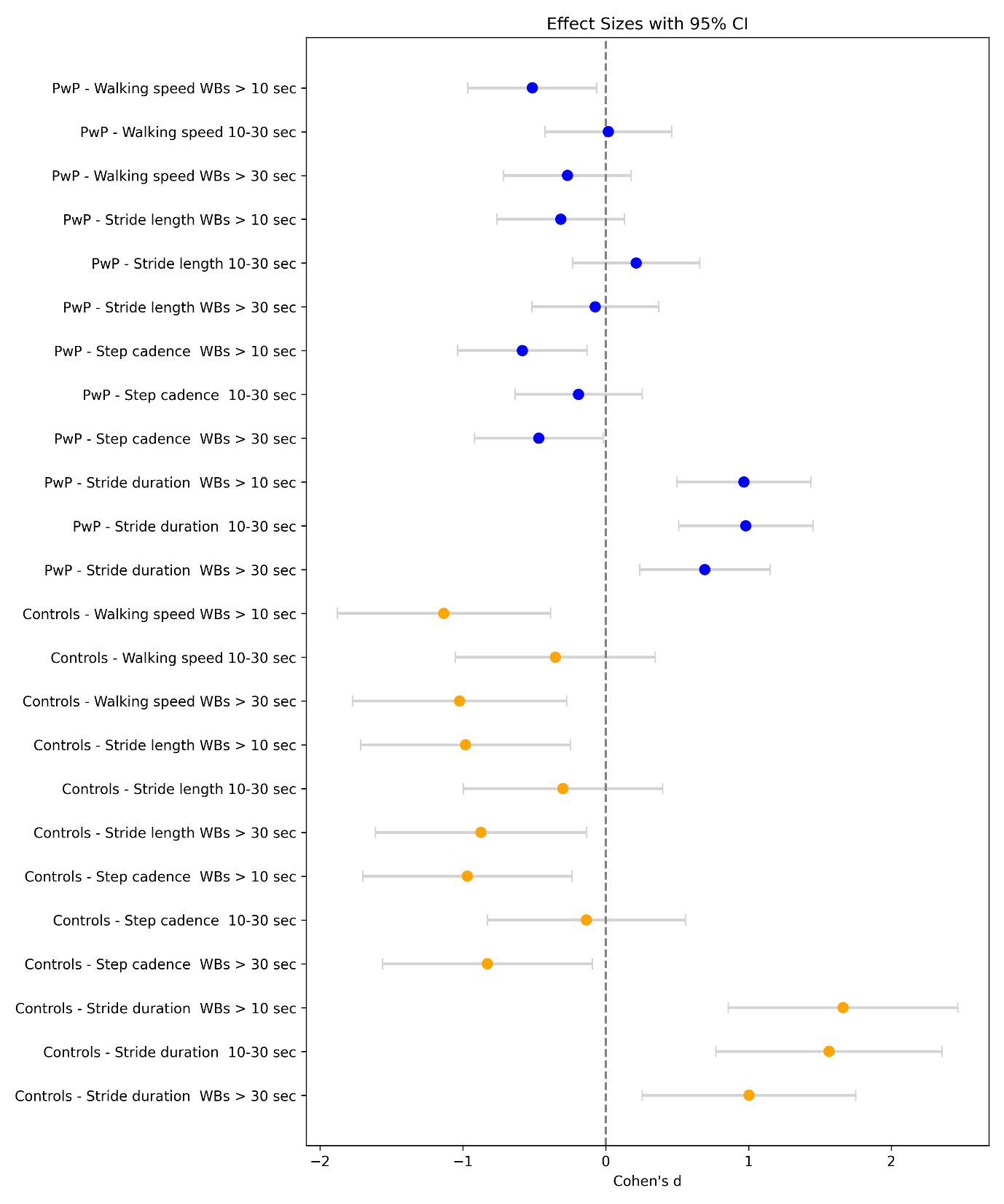
**Supplementary figure 3.** Effect sizes (Cohen’s d) for gait-related digital mobility outcomes compared between indoor and outdoor locations within each cohort. Points represent Cohen’s d values for each variable, with horizontal bars showing 95% confidence intervals. Data are grouped by cohort (People with Parkinson’s (PwP) and controls) and color-coded accordingly.

*
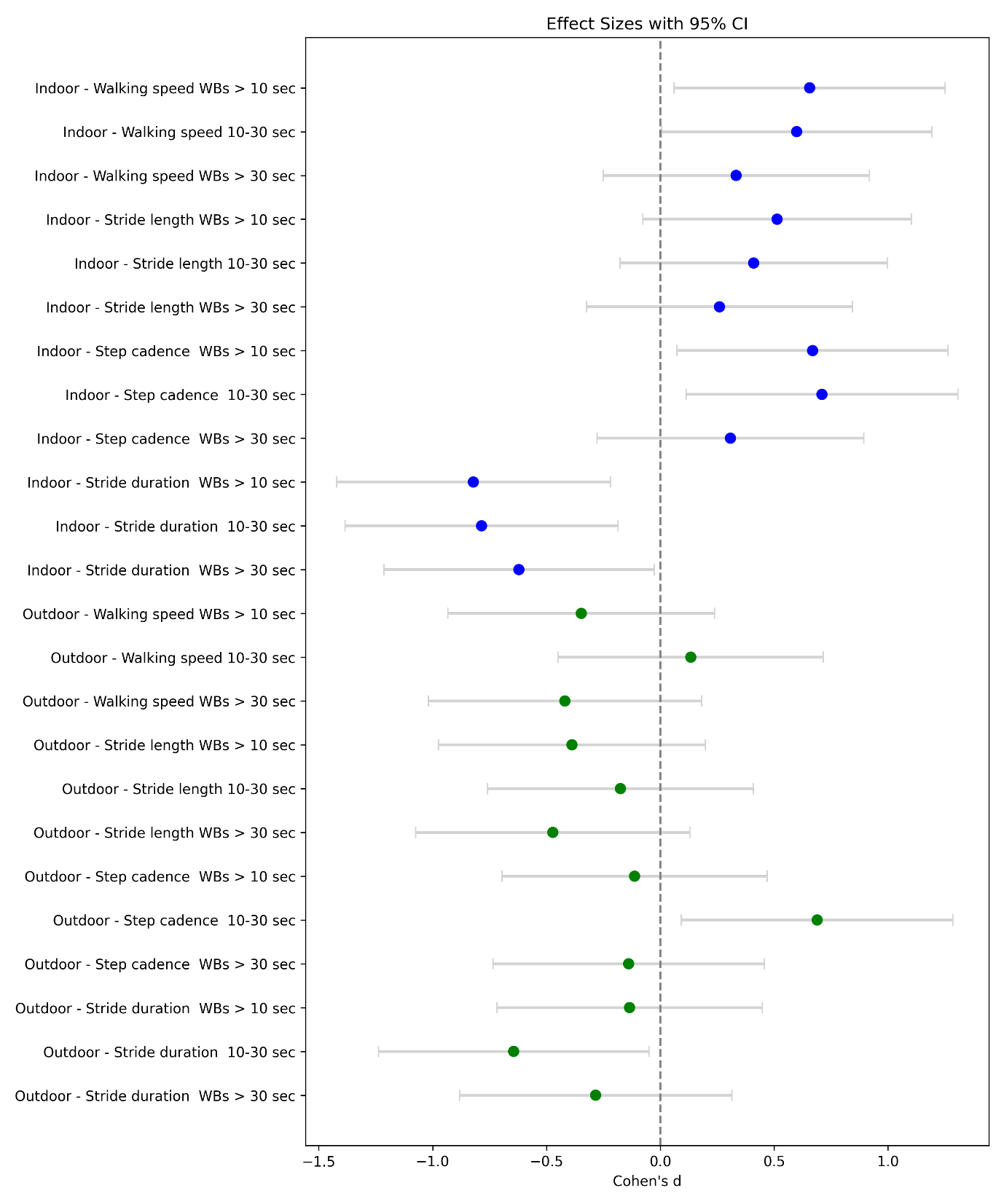
***Supplementary figure 4.** Effect sizes (Cohen’s d) for gait-related digital mobility outcomes compared between each cohort across indoor and outdoor locations. Points represent Cohen’s d values for each variable, with horizontal bars showing 95% confidence intervals. Data are grouped by location and color-coded accordingly.
